# ‘There are a lot of new people in town: but they are here for soccer, not for business’ a qualitative inquiry into the impact of the 2010 soccer world cup on sex work in South Africa

**DOI:** 10.1186/1744-8603-10-45

**Published:** 2014-06-10

**Authors:** Marlise L Richter, Fiona Scorgie, Matthew F Chersich, Stanley Luchters

**Affiliations:** 1 International Centre for Reproductive Health, Department of Obstetrics and Gynaecology, Ghent University, De Pintelaan 185, Ghent 9000, Belgium; 2 African Centre for Migration & Society, University of Witwatersrand, Johannesburg, South Africa; 3 School of Public Health & Family Medicine, University of Cape Town, Cape Town, South Africa; 4 Centre for Health Policy, School of Public Health, University of Witwatersrand, Johannesburg, South Africa; 5 Wits Reproductive Health and HIV Research Unit, Faculty of Health Sciences, University of Witwatersrand, Johannesburg, South Africa; 6 Centre for International Health, Burnet Institute 85 Commercial Road, Melbourne, Victoria 3004, Australia; 7 Department of Epidemiology and Preventive Medicine, School of Public Health and Preventive Medicine, Faculty of Medicine, Nursing and Health Sciences, Monash University, Melbourne, Australia

**Keywords:** Sex work, Prostitution, Sports mega-events, Trafficking, 2010 Soccer World Cup

## Abstract

**Background:**

Sports mega-events have expanded in size, popularity and cost. Fuelled by media speculation and moral panics, myths proliferate about the increase in trafficking into forced prostitution as well as sex work in the run-up to such events. This qualitative enquiry explores the perceptions of male, female and transgender sex workers of the 2010 Soccer World Cup held in South Africa, and the impact it had on their work and private lives.

**Methods:**

A multi-method study design was employed. Data consisted of 14 Focus Group Discussions, 53 sex worker diaries, and responses to two questions in surveys with 1059 male, female and transgender sex workers in three cities.

**Results:**

Overall, a minority of participants noted changes to the sex sector due to the World Cup and nothing emerged on the feared increases in trafficking into forced prostitution. Participants who observed changes in their work mainly described differences, both positive and negative, in working conditions, income and client relations, as well as police harassment. The accounts of changes were heterogeneous - often conflicting in the same research site and across sites.

**Conclusions:**

No major shifts occurred in sex work during the World Cup, and only a few inconsequential changes were noted. Sports mega-events provide strategic opportunities to expand health and human rights programmes to sex workers. The 2010 World Cup missed that opportunity.

## Background

With rapid globalisation, the expansion of technology, and the power and diffusion of different forms of media, international sports tournaments have grown in popularity. Horne and Manzenreiter argue that these tournaments are characterised by two central features: they have a far-reaching impact on the city or country that hosts the event, and they draw considerable media attention [1]. Since the 1980s, international sports events like the Winter and Summer Olympics, FIFA soccer tournaments and the Commonwealth Games have expanded in size, sponsorship and spectators and are now classified as “sports mega-events” [1]. In her research on sport and gender, Theberge traces how sport is traditionally “organised as a male preserve” [2], p.322 while sport advertising and the production of sports mega-events are often tailored specifically to the male spectator, masculine norms and linked to alcohol consumption and sexual titillation [3].

More recently the connection between sports mega-events and sex work has found expression in a “moral panic” about an increase in sex trafficking of women and children into forced prostitution during such times [4,5]. These fears re-surfaced at the 2010 Soccer World Cup (‘2010 WC’), at least partly because sex work and trafficking into forced prostitution were conflated in popular consciousness [6-8].

The nineteenth Fédération Internationale de Football Association (FIFA) Soccer WC took place in South Africa in 2010, the first to be hosted on the African continent. This multi-billion dollar tournament, held every four years, attracts millions of viewers and large corporate sponsorship. It is presented as injecting funds and boosting tourism in the country that wins the bid to host it [9,10]. Following South Africa’s successful bid in 2004, intense media speculation predicted a mass migration of sex workers to South Africa for the 2010 WC, a substantial increase in both sex work and trafficking into forced prostitution, and even a shortage of condoms during the event [11-17]. These predictions were couched within a broader set of concerns about the dangers of the sex sector for the spread of HIV from South Africa into other nations.

During preparations for the 2010 WC, public health researchers and sex work activists warned that similar fears about sex work and trafficking into forced prostitution had circulated before the 2006 WC in Germany and other sports mega-events such as the 2004 Olympic Games, and encouraged the South African government, FIFA and civil society to rather focus on public health and rights-based approaches to sex work [6,18]. Various authors have shown how popular replication of fears about an increase in trafficking into forced prostitution and sex work (and often the conflation thereof) is particularly attractive to sex work abolition groups, anti-immigration lobbies, the media and politicians, and how it does sustained damage to established and evidence-based anti-trafficking programmes [4,19-21]. Table 1 summarises common trafficking into forced prostitution and sex work warnings that have circulated before several sports mega-events, and the evidence that subsequently contradicted these.

**Table 1:** **Myths and facts about trafficking and sex work in the context of sports mega-events (adapted from **[19]**)**

**Event**	**Place**	**Predictions**	**What did research show?**
2012 Summer Olympics	London, United Kingdom	Increase in sex work as well as trafficking of men, women, and children for sex trafficking, forced labor, or both [22,23]	No increase in trafficking - London Met police investigated one case of human trafficking for sexual exploitation [24]
			Majority of female sex workers surveyed say they had fewer customers during the Olympics, and only 7% came to London to seek clients attracted by the Olympics [25]
2010 FIFA World Cup	South Africa	40 000 – 100 000 foreign sex workers or women/children would be trafficked into South Africa and increase in sex work [26,27]	No increase in the supply or demand of sex work during the World Cup [28,29]
			No increase in trafficking – the Department of Justice & Constitutional Development did not find one case of trafficking during the World Cup [30]
2010 Winter Olympics	Vancouver, Canada	Expected increase in trafficking for Olympic Games [31]	No significantly increased odds in reports of new, youth or trafficked sex workers (zero reports) in the Olympic period [32]
2006 FIFA World Cup	Germany	40 000 sex workers/women will be trafficked into Germany for the World Cup [33,34].	No increase in trafficking related to the World Cup [35,36]
			Evidence of 5 cases of trafficking related to the World Cup [37]
			Some sex workers and brothel-owners express disappointment with lack of clientele [38]
2004 Summer Olympics	Athens, Greece	Increase in trafficking of women and children for Olympics [39]	Inconclusive, as no report produced on trafficking and Olympics [35,40]
			An International Organisation for Migration database shows that the organisation assisted 7 victims of trafficking in Athens, but no evidence of links to the Olympics [35]

At the time of the 2010 WC, there was limited international data containing baseline pre-event information on specific sex work settings, and even less on the monitoring of the sex sector over time during a sports mega-event. Our multi-method study aimed to address some of these questions in a South African context.

In 2010, we conducted a quantitative survey of 601 female sex workers before, 508 during and 538 after the WC event. This survey showed no increases in indicators of female sex work supply, including the proportion of female sex workers newly arrived in the city (below 2.5%) or those recently entering sex work (below 1.5%) [28]. Similarly, demand for female sex work, indicated by median number of clients (around 12 per week) and amount charged per transaction ($13) remained stable across the periods pre-, during and post-WC [28]. Self-reported condom-use with clients remained high (more than 92% in all transactions), accessing health care decreased slightly during the event (but was not statistically significant), while about a third of participants across each stage reported some interaction with the police in the month preceding interview. A related study of 663 female sex workers who advertised over the internet and in classified advertisements in three South African cities before, during and after the 2010 WC also found no significant changes to demand and supply of sex work [29].

This paper reports on the qualitative component of the former study and some data from open-ended questions in the survey, which together complements the quantitative findings summed above and broadens the inquiry beyond female sex workers, to male and transgender sex workers.

### Methodology

A team of four academic researchers collaborated with two sex worker NGOs (the Sex Worker Education and Advocacy Taskforce (SWEAT) and Sisonke Sex Worker Movement) to identify three South African cities where Sisonke operates and where 2010 WC matches would be hosted. Johannesburg, the largest city in South Africa, was scheduled to host the most games of any host city. Two sites were selected within Johannesburg: the densely populated, inner-city area of Hillbrow, which has a vibrant, long-standing sex trade [41-43], and Sandton, a wealthy suburb and business district [44]. The second city, Cape Town, was host to the next highest number of WC matches. This coastal city is a popular international tourist destination with a relatively well-documented sex sector [45,46]. The third and final site was a slum area in the predominantly rural North West province; this area adjoined a platinum mine about 15 km outside Rustenburg, the closest city to the soccer stadium where 6 matches were hosted [47].

In this study, sex work was defined as “the exchange of sexual services for financial reward” and male, female and transgender sex workers of 18 years and above were eligible to participate. Data were collected in three phases (before, during and after the 2010 WC), using surveys, presented in detail elsewhere [28,48]. The survey did not recruit a random sample, given that there is no census frame of people in sex work from which to select respondents. This paper includes an analysis of the answers on perceived changes to the sex sector due to the World Cup as provided by male, female and transgender survey participants in the research sites. The primary data source for this article is Focus Group Discussions (FGDs) over these three phases and daily dairies. Table 2 summarises the data analysed for this article.

**Table 2:** Data sources according to research site and period

	Johannesburg^**^			**Cape town**			**Rustenburg**		
	**Surveys**^**#**^	**FGDs**^**^**^	**Diaries**	**Surveys**^**#**^	FGDs^*^	**Diaries**	**Surveys**^**#**^	**FGDs**^**^**^	**Diaries**
Pre-World Cup	-	2	-	-	2	-	-	1	-
World Cup	350 (95%)	2	8	128 (77%)	2	7	123 (91%)	1	8
Post-World Cup	273 (98%)	1	16	83 (83%)	2	6	102 (88%)	1	8

**Johannesburg includes the Sandton and Hillbrow research sites ^#^Brackets indicate the percentage of female participants; FGD Focus Group Discussions; ^^^Female participants only; *One FGD during each phase included females only, and the other male and transgender participants.

Fourteen FGDs with 8-12 participants each focusing on sex work in the context of the World Cup were held. FGD participants – approximately ten participants each in Hillbrow, Cape Town and Rustenburg - were also invited to keep a diary in the language of their choice, over a period of four months. Participants were asked to record, on a daily basis, whether they had had any interaction with the police, with health services and/or with clients, and to describe these interactions and the reasons for them in their diaries. Researchers then collected diary entries from participants in the middle of the 2010 WC and again two and half months after the 2010 WC had ended. Participants were reminded by way of cell phone text messages to complete their diaries and attend scheduled appointments. Researchers endeavoured to retain the same participants for the FGDs and diary component in all three phases, but if participants were absent during scheduled meetings, they were replaced by new participants.

FGDs were held between May and September 2010. This included five FGDs in Hillbrow (two each in the pre- and during-WC period, and one post-WC), three FGDs in Rustenburg (one per phase) and six FGDs in Cape Town (two per phase). All FGD participants were female sex workers, except for FGDs held in Cape Town where approximately half were transgender and male sex workers. Sex work organisations and peer educators recruited sex workers that they knew to participate in the FGDs.

FGDs, moderated by the researchers and translators who made notes during the FGDs, were held in either English or isiZulu in Hillbrow, English in Cape Town and Setswana in Rustenburg. All were audio-taped, transcribed verbatim, and translated into English where appropriate. Participants were encouraged to adopt pseudonyms during the FGDs and these were employed in the analysis. FGD participants were told about the diary and invited to join that component of the study. FGD, diary and survey participation were not linked and overlap between participants is possible.

The University of the Witwatersrand Ethics Committee approved the study (Protocol no. H100304). All participants provided written informed consent to indicate their willingness to participate, and FGD participants provided additional written consent for the FDGs to be audio-taped. FGD participants received a voucher for 100 South African Rand (~US $15) and lunch at the end of each FGD session. No additional reimbursement was given for return of a completed diary.

Data from all three sources (FGDs, diaries and open-ended survey questions) were coded thematically by the principal investigator (MR), and these codes used to build a conceptual framework for further analysis. Findings were compared across cities and different groups of sex workers to determine if any patterns emerged. Results text was then drafted to reflect key themes, and reviewed by all members of the writing team (MR, MFC, FS, SL) for accuracy of interpretation.

## Results

A quarter (17/64) of participants were involved in all three FGDs, close to 40% (25/64) in two FGDs, and a third (22/64) in only 1 FGD (total 64 individuals). Thirty-nine FGD participants accepted the invitation to submit diaries and a total of 53 completed diaries were received: 23 were for the period during the WC and 30 post-WC. Less than half (17/39) of diary participants submitted entries covering both these periods. A total of 322 participants provided a description of the changes they noticed in the sex sector during the WC in the open-ended question in the survey component.

Despite efforts to ensure participant retention in the FGDs and diary components, attrition was high, likely due to heightened scrutiny of the sex sector during the WC period. The sensationalism surrounding the event, the scrutiny of the sex sector, and calls for the registration and mandatory HIV-testing of sex workers in South Africa [49], induced fear and apprehension in the sex worker community [50]. Moreover, at the start of the research project, police raids on the sex sector were taking place and levels of suspicion among sex workers were high.

### Fears, myths and expectations before the WC

In a consultation held in Cape Town in 2009 with civil society and government to forge strategies to deal with the 2010 WC and sex work, sex worker participants expressed fears about an anticipated increase in arrests, sex worker abuse, general crime on the streets and trafficking into forced prostitution during the 2010 WC. Their hopes included increases in the client base, income and foreign currency and that the police would protect sex workers. We explored these issues in FGDs held in the pre-WC period, and found that participants echoed similar sentiments. Some expressed strong trepidation about the start of the 2010 WC, and the persistent and growing threat of police arrests and harassment:

“You see this World Cup is very bad for us, if you are working on the street as sex workers. The problem for us is the police. The police harass us, they collect money from our clients, after collecting the money, they arrest us and then after that you go to the police station. You pay R300. And after R300, tomorrow you are arrested again and again. This work never gains anything. This World Cup is just a World Cup for other people – not for us sex workers” (FGD with female participants, pre-WC, Hillbrow)

Leonie: […] actually, they [the police] want to hide [us] from all the people, not let the people see what is going on in Cape Town. […]

Amber: “[…] the one thing I am worried about and what the cops are constantly telling us, is that quite soon, we are going to pick you up and keep you locked up until the World Cup is over.” (FGD with transgender and male participants, pre-WC, Cape Town)

A number of rumours circulating about the 2010 WC emerged in the data. FGD participants in Rustenburg and Cape Town said they had heard that sex workers from other countries were coming to South Africa for the tournament, thus bringing diseases into the country, such as malaria, swine flu and HIV. One participant in the Rustenburg FGD expressed fears about “people from outside” who, as sex work clients, would “run away” with her, make her a slave or even kill her for body parts. Another warned other FGD participants to look after their children because “people from outside will steal their children”, while a Rustenburg survey participant noted she has been scared of “contracting diseases from foreign countries which we may not have treatment for here in our country”. (40-year old female, post-WC).

Despite these fears, many participants across all FGDs expressed the hope that they would earn substantially more money – and in particular foreign currency – during the 2010 WC, and were excited about the soccer and associated festivities. Some described romantic fantasies of meeting a lifelong partner among the tourists.

Beyoncé: I am going to take off with a nice man. Off to Paris […]

Amber: Ja [yes], what makes me excited is just to know new people. You don’t know if you are gonna meet the man of your dreams, you know, one day. You know what I mean? And then you could just take the chance to take the man of your dreams with the hope that he loves you, the he wants to become mine […] get a life, get married, you know what I mean? A future together – forget about the old future. (FGD with transgender and male participants, pre-WC, Cape Town)

Gilu: Yes, I am excited for the tourists because maybe you can make more bucks. Maybe you meet a client who gives you $100 – You are going to be rich. I am going to be rich […] we are not going to use the Rand [South African currency]. Let me make an example – maybe if you give me $100, I am rich here in South Africa, you see, I am rich. So, I am very excited after July [end of WC], I am going to drive my own car.

Madonna: […] Now if the tourists come to South Africa, maybe 10 out of a 1000 could be taken out of the business [exit from sex work]. That is definitely a step forward for anybody. (FGD with female participants, pre-WC, Cape Town)

### What happened during the 2010 World Cup?

#### No change

During the WC period, survey participants were asked to compare working conditions to the pre-WC period, Similarly, post-WC survey participants were asked to compare working conditions during the WC to the post-WC period. All were asked to describe (in open-ended responses) what changes, if any, they detected. Two thirds (682/1059) of survey respondents believed the sex sector had not been altered during the WC. This view was echoed by the majority of FGD participants, many of whom noted that their expectations had not been fulfilled, or that no differences in their working environment could be detected. FGDs held during and post-WC were often dominated by participants’ expression of intense frustration over the WC’s failure to deliver on their expectations. This was noticeable across all sites.

[…] Facilitator: So the business here wasn’t better or worse than when it wasn’t the World Cup?

Tshidi: According to me, I didn’t see any difference; I think it was just the same (FGD with female participants, during WC, Rustenburg)

There was no changes at all. I am sick and tired of the World Cup. (32-year old female, during WC survey, Cape Town)

In FGD sessions, participants were asked about possible fluctuations in trafficking into forced prostitution during the 2010 WC. Participants doubted that such increases had occurred:

Facilitator: Remember the last time we spoke about trafficking, people [FGD participants] were saying they were scared that the clients might come and traffick them to other countries. Have you seen any trafficking, have you heard people talk about trafficking?

Nthabi: No, we didn’t hear anything about that. (FGD with female participants, during WC, Rustenburg)

In addition, none of the respondents in the survey mentioned trafficking into forced prostitution.

While the perception of ‘no change’ dominated overall, there were those who felt the WC had indeed brought about noticeable change., participants were divided about whether WC-related changes were “good” or “bad” overall; no patterns in these perceptions were noted between sites. We separated descriptions of changes into two broad themes relating to changes in work conditions, income and client relations, and to interaction with the police, including police harassment.

#### Negative changes in working conditions, income and client relations

Several sex workers in the FGDs and surveys felt that the WC had been detrimental to their business and income:

I think the World Cup brought poverty. From the eleventh [of June – the start of the WC] I didn’t get anything. I was used to deposit R700 [$93] every Monday, unless if I was going to use it, but from when the World Cup started, I only managed to save R150 [$20]. (FGD with female participants, during WC, Hillbrow)

Me, I feel happy for the World Cup to be finished, because during the World Cup I didn’t even have a single cent because the police are after us and I didn’t work well. (FGD with female participants, post-WC, Hillbrow)

“There are a lot of new people in town. But they are here for soccer not for business” (24-year old female, during WC survey, Cape Town)

Across research sites, some participants reported a decrease in their regular clientele:

I have said there is no money for us in the World Cup so my regular clients don’t come out as usual. Like this week - no money. I have been sitting the whole week on dead coal, sorry for me. (FGD with transgender and male sex workers, during WC, Cape Town)

I used to have many clients of truck drivers who are from Zambia and Durban, but now, since this World Cup period, they don’t even phone me and they all stopped both sides. (32-year old female, during WC survey, Hillbrow)

“There’s no business at all, it’s too quiet. We are not seeing what we were expecting. Our clients are broke and busy watching soccer” (33-year old female, during WC phase, Hillbrow)

One participant from Cape Town noted that clients from other countries were not as exotic as originally anticipated:

Well there is nothing special about these foreign people. The way they are, it’s just still the same. Like me and you, because it’s just that they are from another country and they are abroad. There is nothing special about them, but the only thing is just you get the same money and less clientele. (FGD with transgender and male participants, during WC, Cape Town)

#### Positive changes in working conditions, income and client relations

For some participants across all research sites, however, business had picked up during the WC and may have improved because payment had been made in foreign currency.

Sometimes clients pay 100 Dollar or Euros. Business is been really good. I wish the World Cup would return one day. (23-year old female, during WC survey, Hillbrow)

Most of the foreign clients they like black women and I got foreign currency first [time] in my life. (43-year old female, during WC survey, Cape Town)

The foreigners are gone with their money. I went out with a few from overseas. Now there’s a difference ‘coz they’re gone with their money. (32-year old transgender participant, post-WC survey, Sandton)

A number of participants reported that foreign clients had paid more, or were more courteous than their regular clients. One woman attributed higher payments from regular clients to this apparent competition from new clients:

I met clients that treated me very differently comparing to those from here. They never treated me like a prostitute. They were very kind. (25-year old female, during WC survey, Cape Town)

Also the regulars, it’s almost like they were afraid that the foreigners would get more from us or something like that, and so they would pay more, but still we did our business. It was quite good. For me, it was quite worth it. (FGD with female participants, during WC, Cape Town)

For a small number of participants, longer working relationships had been established with some clients, which were lucrative:

At least I found a man I stay with the whole two weeks. Then he gave me $800, so it was better for me during the World Cup. (35-year old female, post-WC survey, Sandton)

15 July 2010: Wake up, eat breakfast, clean my place, meet a client for a sleep-over. We went shopping. He was a German. We went for lunch. He took me to a club, we had drinks. We went to his hotel and I sleep over. Next morning he gave me R1000 [$130]. […]

29 August 2010: This was the last day for my German client. We spent it at my place. I cook dinner for him. I make it very special. I went with him to the airport. He left me a R15 000 [$2000]. He asked me not to prostitute again. (Diary entry, Cape Town participant)

Participants also noted the benefits of expanding their social and clientele networks, while frequent reference was made to receiving gifts from clients.

I was able to meet different people from other countries. We even exchanged phone numbers. It was very exciting; we go out for dinner and drinking. (35-year old male, during WC phase survey, Cape Town)

The people I sold to, one of them bought me a ticket to go to the stadium to watch soccer. It was my first time to watch soccer. I don’t even wish for that day to pass. (24-year old female, during WC phase survey, Rustenburg)

A spectrum of responses from survey participants described improved working conditions brought about by the WC. These included the upgrading of hotels (popular solicitation venues in Johannesburg), better hotel security (private security hired by hotel management to provide a secure environment for sex workers and clients), less criminal activity, and overall goodwill generated by the festive atmosphere of the 2010 WC.

The hotels have been renovated and there is more security than before. (34 year old female, post-WC, Hillbrow)

Business is much better than before and criminals are also quiet. Because of soccer we don’t fear anymore. (24-year old female, during WC phase, Sandton)

#### Sex workers explain changes to the sex sector attributed to the 2010 WC

One of the most notable findings was that participants had developed a complex web of reasons to account for how the 2010 WC had affected business. Many participants attributed the decline in client numbers to the public’s absorption with soccer:

As for me, there was no business for me, no clients. People were watching ball - they don’t want to buy us. (38-year old female sex worker, post-WC survey, Hillbrow)

The other thing that I have noticed, especially for us who work at the streets, at the time we’re going to work, it is exactly the time all these men are sitting in the bars watching soccer, and you would find that there is no-one at the streets for our services, you see. (FGD with female participants, during WC, Hillbrow)

In contrast to the street-based sex worker’s observation above, soccer matches were also said to have drawn more people into public entertainment areas where indoors-based sex workers operated, and thus increased their clientele.

We made good bucks because in taverns there is a big screen TV, so lots of people were there. (29-year old female, post-WC survey, Cape Town)”Business was so fast and clients were so many and some clients would watch ball [soccer] inside the hotel [where she worked]” (30-year old female, post-WC survey, Hillbrow)

The cold weather in Johannesburg was cited as an inhibitor for clients, and was cited as a particular problem for sex workers who solicited outside in this city:

[It] is Summer now - we are getting clients now a little bit. The World Cup was done in Winter so many guys don’t go out. (FGD with female participants, post- WC, Hillbrow)

Yes there is change. It is cold and we stand on the streets cold as it is, and leave without money. This soccer came with bad luck. (24-year old female, during WC phase, Hillbrow)

Other participants saw the demand for their services fluctuate in line with the victories or losses of clients’ favourite teams.

Clients are buying us if their team does not lose. (25-year old female, during WC survey, Sandton)

2 July 2010: Clients not happy with Soccer World Cup results - not buying. (Diary entry of Hillbrow-based participant)

Some participants attributed lack of client interest to the widely disseminated pre-WC warnings for tourists about the dangers of South Africa (including HIV transmission) and the threat of police.

Milo: …those white people, blacks who are coming from England everywhere, but to buy you beer […]. They are still afraid just because they heard that in South Africa, it’s too dangerous, you understand, so they are scared to go with us, you understand? Sometimes, you know, they are just scared they say “No, let me buy you beers.” (FGD with female participants, during WC, Hillbrow)

Interviewer: And you my sister what were you saying? You were saying the World Cup is not treating you right?

Sisi: No – not at all because everybody is scared. There were lots of threats especially with the sex worker because the police were telling us ‘We are going to get you’ and stuff like ‘We don’t need you at the World Cup’. So, some of the people they were very scared. Every time you are in a hotel and police just came in and people [clients] they get scared and they just walk out (FGD with female participants, during WC, Hillbrow)

Participants in Cape Town in the survey and FGD components attributed the drop in business to the arrival of new sex workers in their area, although participants in Hillbrow insisted they had observed no newcomers.

I saw a lot of new faces [new sex workers] around. There was competition - everyone wanted to benefit. (24-year old female, post-WC survey, Cape Town)

In my area there were new ladies from Mozambique, Tanzania, Zambia and Congolese - yeah there were new ladies. (FGD with female participants, during WC phase, Cape Town)

Shingi: We heard that there are 40 000 bitches coming here.

Facilitator: Do you think that affected your work?

Tebogo: I think there is no one who came here. (FGD with female participants, during WC phase, Hillbrow)

The fact that many soccer spectators viewed the 2010 WC festivities as a family activity was widely commented on; this precluded clients from seeking out their services.

The World Cup brought hunger to me. The clients used to call me and collect me, but now they are no longer coming. When I call them I get the voicemail or they will tell me that they are busy with their families. (45-year old female, during WC phase, Cape Town)

*The people from outside did not buy us. They said they came with their partners. (32-year old female, during WC phase, Rustenburg*)

#### Contact with police and police harassment

No consensus emerged on whether police harassment differed during the World Cup in comparison to other times. Participants in Cape Town and Johannesburg remarked on the increased visibility of police, private security at malls, and police volunteers, but not those in Rustenburg. Yet, within all three data sources, the constant threat posed by the police to sex worker private life and work was unmistakable. Many participants described during and post-World Cup being recently arrested or efforts to avoid arrest or harassment, whether working or not, often by paying bribes to the police. One of the Hillbrow-based diary participants documented how she was arrested six times in the month of July 2010. Participants frequently described extortion, unwarranted violence and humiliation by the police.

They [the police] arrived where we [sex workers] are working from, when they got there the client was on top of me […] The client was scared already because they were already here threatening to arrest us, so they said to him “Continue doing what you’ve been doing”. He did it in front of them, watching. Then they beat him up so bad. Then they pulled him down the stairs and they took his money, they told him that if he doesn’t give them the money they will arrest him, so he had to take all the money he had in the wallet and gave it to them. They went and left him like that with nothing on him (FGD with female participants, during WC, Hillbrow)

The police harassment is too much. Every day they disturb us asking many questions and use [a] spray gun to [pepper]spray us while we are walking (29-year old female, during WC survey, Hillbrow)

The phenomenon of police finding condoms on suspected sex workers and using this as “evidence” that they were selling sex or using drugs, was mentioned by a few participants:

They [the police], when they see me, they are like “Come here” […] and they search my bag in front of everybody and they are like “What?” and they found condoms in my bag. They say to me “I don’t want to see you with condoms in your bag. What do you use it for? You use it to do drugs, to do heroin” and he says “I am going to lock you up when I see condoms in your bag. Don’t put condoms in your bag”. Then I am like “What? You can’t tell me not to put condoms in my bag, I am going to get me ten packs tomorrow.” (FGD with transgender and male participants, during WC, Cape Town)

“[…] if you have condoms in your bag, if they [the police] suspect that you are a sex worker, and they found condoms in your bag, they will arrest you because you are a sex worker. So, which means people who are carrying condoms are only sex workers. It is so wrong. (FGD with female participants, during WC, Cape Town)

In contrast to those who noticed increased police surveillance of sex workers, a number of participants in Johannesburg thought that police arrests and harassment had lessened during the 2010 WC. This makes it difficult to draw conclusions about whether there were differences between police actions between the cities studied.

Well it is boring, there is no money, as there is no money…Often we get threatened by the police, but lately it has been quiet since they are also watching soccer, or maybe they are on guard at the stadiums. They are not as they used to be before the World Cup started. As we told you when we started with this research that they [the police] told us that they are going to arrest us should they find us on the street, but now it has been quiet, but maybe they do arrest when I’m not there, but since the World Cup has started I haven’t been arrested. (FGD with female participants, during WC, Hillbrow)

During the World Cup period, police were not after us, and it was not busy. Right now police are after us - end of the month, Friday, Saturday. It’s our busy days - (24-year old female, post-WC survey, Sandton)

Although rare, a few participants even noted that some police had appeared friendly and non-intimidating.

19 July 2010: two police were friendly. We were running away when we saw the [police] van. They said no, we are not those police who abuse you. They were not coming to arrest. (Diary of a Hillbrow-based participant)

## Discussion

Data from the survey component provides a key point for this discussion: two thirds of male, female and transgender participants surveyed noted no change in the sex industry during the WC. Survey participants knew the sex sector well – about 40% (405/979) had been in the sex sector for more than 5 years while around 20% (177/979) had been sex workers for less than a year (data not shown). Sex workers themselves are therefore best placed to assess whether the sex sector had changed due to external influences such as a sports mega-event. Our survey data show that changes were experienced by only a third of survey participants, and among these, descriptions were often related to frustrated expectations of what the WC could have meant in terms of clients and financial enrichment. These were possibly unrealistic hopes, fuelled by the media’s fascination with sex work.

Participants in all three data sources seldom mentioned an increase of sex workers in the places they were working – whether new or migrant– or noticed any suspected trafficking into forced prostitution. It has been argued elsewhere that sex workers are best placed to identify victims of trafficking into forced prostitution [51]. The lack of evidence of trafficking in this study, together with the fact that South Africa’s Department of Justice & Constitutional Development did not find any cases of trafficking into forced prostitution during the World Cup [30] strengthens the claim that widespread fears about trafficking constituted a ‘moral panic’ as it did during other sports mega-events [4,7,52]. This emergence of moral panics over sex at key points in South Africa’s history has also been documented elsewhere [4,53].

The descriptions provided by the minority of participants who noticed a change in the sex sector offer a snapshot of the varied nature of the sex sector in the research sites in 2010. Accounts of the changes noted are heterogeneous - often conflicting in the same research site and across sites. Structural factors that are detrimental to sex workers’ ability to keep themselves and others safe, to work in a secure environment and to make a dignified living, surfaced strongly in sex worker descriptions of their daily work conditions. These factors were in place prior to, during and post-WC, and sadly continue to define sex workers’ lives in this region. The threat, and materiality, of police abuse was manifest but there is no conclusive evidence that this increased during the World Cup. Yet the ongoing criminalisation of sex work, the wide spectrum of power this confers on police and others, and their subsequent abuse thereof, as also documented elsewhere [55-57], emerged from the narratives and is a point of particular concern. Regrettably, the South African government did not take advantage of the 2010 WC as an opportunity to introduce public health-centred interventions to improve the well-being and safety of sex workers [6].

The study has a number of limitations. Research sites were purposively selected, based on the presence of sex worker advocacy groups and peer education work. The survey component used a non-random sampling design that is influenced by the social networks of seeds [57] while the FGD and diary components relied on sex worker peer educators or sex workers known to the collaborating NGOs. Sex workers who did not work, or engage, with other sex workers or sex work NGOs may not have been reached, given the reliance on social networks inherent in the sampling methods used. This means that new sex workers who did not have the chance, or resisted, networking with an established sex worker community may have been overlooked and that their perspectives were not included in this study. Similarly, it could be contended that victims of trafficking into forced prostitution may have been so concealed that they were not noticed by sex worker study participants. Conversely, if the former were so hidden, it is unlikely that potential clients would be able to access them. Data on socio-demographic characteristics of the FGD and diary participants were incompletely captured, this weakens the ability to interpret the study results.

In conclusion, significant tabloid space, speculation, funds and anxiety were expended on fears about increases in trafficking into forced prostitution and sex work during the 2010 WC. These efforts could have been better spent tackling the root causes of vulnerability in sex work settings, the material conditions of sex workers, and the socio-legal factors that foster and support police violence and abuse of sex workers [58]. It would thus be prudent for future campaigns and policy work on sports mega-event, sex work and human rights to focus on the less sensational, but more pressing aspects of the sex sector; on the structural factors that constrain sex worker health, safety and livelihoods on a daily basis.

## Competing interests

The authors declare that they have no competing interests.

## Authors’ contributions

MR led the design of the study, trained interviewers, coordinated data collection, performed the thematic analysis, wrote the first draft and edited subsequent drafts of the manuscript. FS, SL and MFC gave input into study design, data collection and analysis, helped draft and edit the manuscript. All authors read, gave substantial input and approved the final manuscript.
